# Analysis of the incidence and influencing factors associated with binary restenosis of target lesions after drug-coated balloon angioplasty for patients with in-stent restenosis

**DOI:** 10.1186/s12872-022-02923-z

**Published:** 2022-11-20

**Authors:** Weihao Xue, Jun Ma, Xiaojie Yu, Zhisheng Ruan, Yuanxue Sun, Tianbo Wu, Xinmin Zhang, Lianpin Wu

**Affiliations:** Department of Cardiology, The Second Affiliated Hospital and Yuying Children’s Hospital of Wenzhou Medical University, Wenzhou, 325027 China

**Keywords:** Coronary artery disease, Drug-coated balloon, In-stent restenosis, Influencing factors

## Abstract

**Background:**

Drug-coated balloon (DCB) is a novel and effective device for coronary artery disease patients with in-stent restenosis (ISR). However, the incidence and possible influencing factors associated with binary restenosis have not yet been adequately assessed.

**Methods:**

The data are extracted from a prospective, multicenter, randomized controlled trial. A total of 211 patients with ISR were enrolled at 13 centers from August 2017 to October 2018 and treated with DCB. At the 9-month coronary angiographic follow-up, patients were divided into restenosis and non-restenosis groups, and demographic data, lesion features, and laboratory tests were retrospectively reviewed. Furthermore, logistic regression analysis was used to identify possible influencing factors.

**Results:**

All patients successfully underwent treatment, and 166 patients with 190 lesions took part in angiography follow-ups at 9 months. Of these, 41 patients with 44 target lesions developed restenosis following treatment, and the incidence of ISR was 24.7%. There were significant differences in the average length of target lesions and the number of multivessel lesions and fasting plasma glucose (FBG) between the two groups (*p* < 0.05). Demographic data, cardiac risk factors, left ventricular ejection fractions (LVEF), blood routine tests, biochemical tests, and other features of devices and lesions showed no difference. Logistic regression analyses showed that FBG > 6.1 mmol/L (OR: 7.185 95% CI: 2.939–17.567 *P* < 0.001) and length of lesion (OR:1.046 95% CI: 1.001–1.093 *P* = 0.046) were associated risk factors.

**Conclusions:**

The longer length of lesions, more target lesions and FBG > 6.1 mmol/L per individual may be characteristics of patients showing ISR following treatment. Studies with larger sample size, and more complete follow-up data are needed in the future to expend on these findings.

**Trial registration:**

No.: NCT04213378, first posted date (30/12/2019).

## Background

In-stent restenosis is a common complication of percutaneous coronary intervention (PCI) for coronary heart disease which is defined as a stenosis ≥50% of the lumen diameter within a coronary stent or up to 5 mm from the stent edges (by the angiographic definition) [1]. The primary etiology of ISR is usually related to operator technique, the characteristics of the stent and patient- and biologically related conditions [2]. Therefore, solving how to effectively treat ISR has become an urgent problem in the medical community. Initially, plain old balloon angioplasty was used to treat ISR. However, restenosis occurs at a high rate of 40 to 50% [3]. Stent implantation again has been confirmed to be an effective method by using bare-metal stents (BMS) or drug-eluting stents (DES). Notably, some studies have demonstrated that the effect of DES re-implantation is superior to that of simple balloon angioplasty and balloon cutting [4]. The use of DES, especially second-generation DES, has further reduced restenosis rate. Unfortunately, the target lesion revascularization (TLR) incidence is approximately 10 to 20% at 5 years [5–7]..

Drug-coated balloon is a novel device that combines a balloon with a drug to treat coronary artery lesions, dilate target arteries, and release anti-proliferative drugs such as paclitaxel to inhibit endangium hyperplasia by leaving no metal behind [8]. In 2006, the first worldwide randomized and multicenter clinical trial studying the treatment benefits of DCB in-stent restenosis found that DCB was superior to conventional balloon treatment for BMS-ISR with a lower incidence of re-restenosis and major adverse cardiovascular events (MACE) [9]. A meta-analysis of 24 randomized controlled trials involving 4880 patients showed that DCB and DES had superior clinical outcomes for ISR compared with other current interventional therapies [10]. Furthermore, the 2018 ESC/EACTS guidelines have mentioned several advantages of DCB treatment and recommended its use in in-stent restenosis as a Class Ia device [11]. However, some patients still develop binary restenosis after drug-coated balloon therapy, and there are not enough studies on the risk of re-restenosis after ISR treatment with DCB. Therefore, we conducted this study to investigate the incidence and possible influencing factors associated with target lesion re-restenosis after DCB angioplasty in patients with ISR.

## Method

### Study design and population

**Fig. 1:**
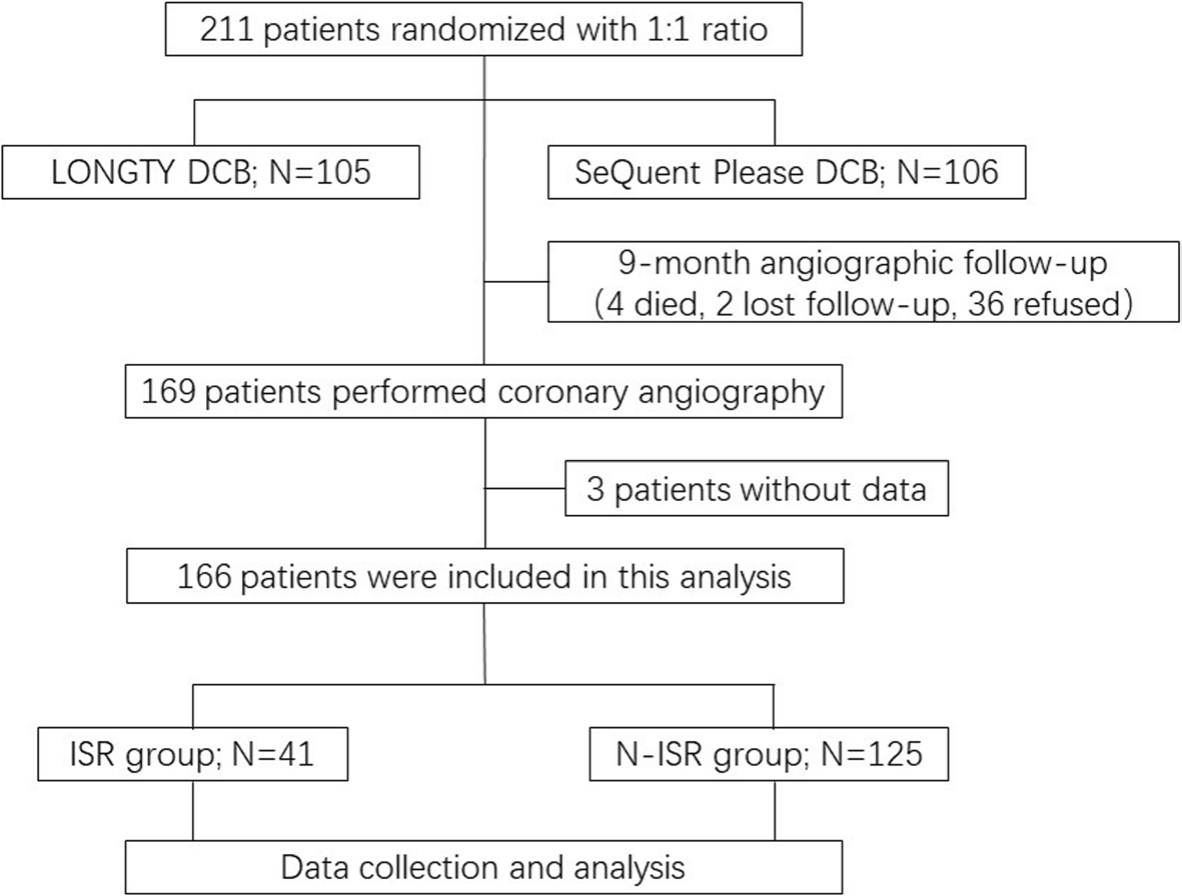
Study flow

From August 2017 to October 2018, 211 patients diagnosed with ISR of coronary artery disease (CAD) were enrolled from 13 centers and randomly allocated in a 1:1 ratio to be treated with a drug-coated balloon (LONGTY DCB or SeQuent Please DCB). After an average of 9 months of angiographic follow-up, they were divided into the restenosis and non-restenosis groups based on the presence of binary restenosis. The study flow diagram is shown in Fig. 1.

The major inclusion criteria were the following: patients aged between 18 and 80 years; patients with stable angina pectoris, or unstable angina pectoris, history of myocardial infarction or evidence of asymptomatic ischemia; in-stent restenosis occurring in de-novo coronary artery for the first time; Mehran type I–III; LVEF> 30%; reference vessel diameter ranging from 2.0 to 4.0 mm, lesion length ≤ 30 mm, diameter stenosis ≥70% or ≥ 50% with evidence of ischemia and < 30% after pre-procedure with dissection type ≤ B; other lesions more than 10 mm away from the target lesion; patients receiving successful treatment and followed up with coronary angiography after 9 months and the key data had been accurately recorded and saved. The exclusion criteria were as follows: patients with symptoms of ST-elevation myocardial infarction (STEMI)/Non-STEMI (NSTEMI), fitting in with the typical change of electrocardiogram (ECG) or myocardial enzyme, intolerant to aspirin or clopidogrel therapy, or having active gastrointestinal ulcers, stroke/ transient ischemic attack within 3 months, severe heart failure (NYHA IV), renal failure, valvular heart disease, cardiac shock, hemodynamic instability, or patients with refractory ventricular arrhythmias life expectancy with less than 12 months life expectancy were not enrolled in this study. Additionally, patients with extensive thrombus in the target vessel, total occlusion within a grade of TIMI 0 (Mehran IV), bifurcation lesions treated by two stents, more than 3 target lesions or 2 major epicardial vessels, and patients requiring more than one balloon to treat a single lesion were excluded. Furthermore, patients with unretained coronary angiography images or missing key data were not included.

New ethics approval was not applicable, because the original study had obtained ethical approval and informed consent in all participating centers when conducting the study, and the study was conducted in accordance with the principles of the Declaration of Helsinki.

### Devices, intervention protocol, and data management

We use two different DCBs in this study. Both SeQuent Please DCB (B. Braun, Melsungen, Germany) and LONGTY DCB (Barty Medical, China) are coated with paclitaxel 3 μg /mm2 of the balloon surface. The balloon length ranged from 10 to 30 mm, and the diameter was 2.0–4.0 mm. All patients were treated with aspirin and clopidogrel (or ticagrelor) and received a loading a dose of clopidogrel (300–600 mg), ticagrelor (180 mg), or aspirin (100 mg/day for 3 days or one dose of 300 mg) before surgery. During the procedures, full anticoagulation was obtained with an initial bolus of 100 U/kg of unfractionated heparin (additional boluses were administered as required to achieve an activated clotting time > 300 s).

To achieve ≤30% stenosis, all patients received pre-dilation treatment, then they were randomly allocated in a 1:1 ratio to be treated with LONGTY DCB or SeQuent Please DCB. The device’s size depended on the operator’s decision, and post-dilation was performed if the result of dilation was not satisfactory. All patients were required to complete various tests before surgery, and relevant data were recorded and stored in the database, including demographic characteristics (age, sex, BMI), risk factors (history of smoking, diabetes, hypertension, hyperlipidemia, myocardial infarction, heart failure, myocardial ischemia), information of target lesion and operating equipment and other key information. All patients were followed up at 1, 6, and 12 months by telephone, mail or in the ward and performed coronary angiography again to evaluate the lumen diameter stenosis at 9 months.

### Statistical analysis

All statistical analyses were performed using SPSS 26.0. The categorical variables were expressed as count (percentage) and analyzed using the χ^2^ test or Fisher’s exact test, variables were displayed as mean ± standard deviation (SD) and analyzed using Student’s t-test, and *P*-values < 0.05 were considered statistically significant, and logistic regression analysis further screened the variables with P-value < 0.05. A small number of missing data (Weight data were missing in 5 patients, height data were missing in 14 patients, and blood routine data were missing in 3 patients) are replaced using the “Replace Missing Values” function by SPSS for further analysis.

## Results

### Comparison of general clinical data between the two groups (cardiovascular risk factors, demographic characteristics)

Excluding 4 patients who died, 2 patients who were lost to follow-up, and 36 patients who refused to undergo coronary angiography, a total of 169 patients (78.7%) participated in angiographic follow-up after an average of 9 months (9.25 ± 3.94). Of these 169 patients, 3 without accurate data were excluded, and 166 were included in the analysis. No significant difference was noted in clinical features between patients who underwent follow-up angiography and those who did not. After statistical analysis, 41 were diagnosed with ISR, and the incidence of ISR was 24.7%. The comparison of baseline characteristics between the two groups is shown in Table 1. As presented, there were no significant differences in the demographic characteristics like age and BMI between the ISR group and non-ISR group (all *p* > 0.05). In contrast, the percentage of men in the ISR group was lower than another one (65.9% vs 80.0%, *P* = 0.064). Patients with multivessel disease were more likely to develop ISR than patients with single vessel lesion (22% vs 9.6%, *P* = 0.039). Other general baseline characteristics including smoking history, hypertension, hyperlipidemia, diabetes mellitus, history of myocardial infarction, history of heart failure, and history of myocardial ischemia, were not significantly different between the two groups (all *p* > 0.05).

**Table 1:** Comparison of baseline data between ISR and non-ISR groups

	ISR group	N-ISR group	t/χ^2^	*P*
Age, year	61.5 ± 10.5	62.4 ± 8.4	−0.459	0.648
Male sex, n (%)	27 (65.9%)	100 (80.0%)	3.437	0.064
BMI	24.59 ± 3.81	24.81 ± 3.04	−0.339	0.648
Smoking history, n (%)	14 (34.1%)	54 (43.22%)	1.046	0.306
Diabetes mellitus, n (%)	17 (41.5%)	52 (41.6%)	< 0.001	0.988
Hypertension, n (%)	24 (58.5%)	86 (68.8%)	1.455	0.228
Hyperlipidemia, n (%)	4 (9.8%)	27 (21.6%)	2.852	0.091
Previous MI, n (%)	10 (24.4%)	45 (36.0%)	1.878	0.171
Previous HF, n (%)	12 (23.5%)	27 (21.6%)	0.078	0.780
Myocardial ischemia, n (%)	5 (12.2%)	25 (20.0%)	1.270	0.260
LVEF	58.27 ± 8.85	60.67 ± 8.74	−1.521	0.130
Clinical features, n (%)			5.523	0.063
Silent ischemia	10 (24.4%)	33 (26.4%)		
Stable angina	17 (41.5%)	29 (23.2%)		
Unstable angina	14 (34.1%)	63 (50.4%)		
Target lesions, n (%)				
One	32 (78%)	113 (90.4%)		0.039
Two or more	9 (22%)	12 (9.6%)		

Data are presented as mean ± standard deviation or n (%)

*BMI* Body mass index, *MI* Myocardial infarction, *HF* Heart failure, *LVEF* Left ventricular ejection fraction

### Laboratory indicators

We analyzed the results of laboratory tests in both two groups of patients. The results are summarized in Table 2. As demonstrated, the percentage of patients with FBG > 6.1 mmol/L in the ISR group reached 82.9% compared with 39.2% in the N-ISR group (*P* < 0.001). Additionally, the WBC, neutrophils, RBC, hemoglobin and platelet count did not differ. For biochemical indexes, the level of ALT, AST, ALP, TBIL, DBIL, TG, TC, LDL-C, HDL-C, and Cr also had no significant difference between the two groups (all *P* > 0.05). Overall, the laboratory test results did not show a significant difference between the two groups of patients.

**Table 2:** Laboratory tests

	ISR group	N-ISR group	t/χ^2^	*P*
WBC,10^9/L	6.69 ± 1.91	6.76 ± 1.92	−0.198	0.843
N, 10^9/L	4.34 ± 1.68	5.13 ± 7.26	−1.126	0.262
RBC, 10^12/L	4.50 ± 0.62	4.58 ± 0.50	−0.779	0.493
Hb, g/L	134.88 ± 15.85	140.09 ± 15.66	−1.843	0.067
Plt, 10^9/L	223.41 ± 67.44	209.94 ± 60.24	1.139	0.259
ALT, U/L	30.51 ± 29.70	32.96 ± 36.87	−0.431	0.668
AST, U/L	26.71 ± 17.65	28.98 ± 29.51	−0.595	0.553
ALP, U/L	83.75 ± 38.41	81.66 ± 24.72	0.327	0.745
TBIL, **μ**mol/L	12.91 ± 5.95	12.31 ± 4.97	0.584	0.562
DBIL, **μ**mol/L	4.06 ± 3.26	3.98 ± 1.92	0.148	0.883
Cholesterol, mmol/L	3.94 ± 1.13	3.96 ± 1.51	−0.095	0.924
TG, mmol/L	1.55 ± 0.86	1.95 ± 1.81	−1.889	0.061
HDL-C, mmol/L	1.09 ± 0.30	1.06 ± 0.23	0.644	0.522
LDL-C, mmol/L	2.10 ± 0.85	2.01 ± 0.81	0.550	0.584
Cr, mmol/L	71.95 ± 22.85	76.31 ± 22.43	−1.067	0.290
Glu > 6.1, mmol/L	34 (82.9%)	49 (39.2%)	23.612	< 0.001

Data are presented as mean ± standard deviation or n (%)

*WBC* White blood cell, *N* Neutrophils, *RBC* Red blood cell, Hb Hemoglobin, *Plt* Platelet, *ALT* Alanine aminotransferase, *AST* Aspartate aminotransaminase, *ALP* Alkaline phosphatase, *TBIL* Total bilirubin, *DBIL* Direct Bilirubin, *TG* Triglycerides, *HDL-C* High-density lipoprotein cholesterol, *LDL-C* Low-density lipoprotein cholesterol, *Cr* Creatinine, *Glu* Glucose

### Target lesion characteristics and procedure information

The lesion and device characteristics were analyzed per lesion. From the target lesion data of two groups of patients in Table 3, we found that the average length of target lesions in the ISR group was much longer compared with the non-ISR group (20.26 ± 7.12 vs 17.19 ± 7.50, *P* = 0.023). As for the average diameter stenosis, the number of calcified lesions, the reference vessel diameter, and the location of target lesions before drug-coated balloon therapy, there was no significant difference between the two groups (*p* > 0.05). All patients had received pre-dilation, and there was no correlation between the number of pre-dilated balloons used in each lesion and the appearance of restenosis.

**Table 3:** Lesions and devices characteristic between two groups

	ISR group	N-ISR group	t/χ^2^	*P*
Target lesions				
Diameter stenosis, %	84.5 ± 10	82.9 ± 10	0.912	0.365
Lesion length, mm	20.26 ± 7.16	17.35 ± 7.70	2.324	0.023
Reference vessel diameter, mm	3.45 ± 3.35	2.97 ± 0.39	0.948	0.348
Calcified lesions, n (%)	9 (20.5%)	19 (13.0%)	1.490	0.222
Location of arteries, n (%)			2.956	0.399
LAD	26 (59.1%)	68 (46.6%)		
LCX	6 (13.6%)	20 (13.7%)		
RCA	11 (25.0%)	56 (38.4%)		
D/RI	1 (2.3%)	2 (1.4%)		
Pre-dilation balloon				
Number in each lesion, n (%)			0.531	0.466
One	20 (45.5%)	75 (51.7%)		
two or more	24 (54.5%)	70 (48.3%)		
Treating balloon				
Diameter, mm	2.91 ± 0.35	2.99 ± 0.38	−1.289	0.199
Length, mm	23.44 ± 5.19	21.99 ± 5.21	1.604	0.110
Duration of inflation, s	58.07 ± 11.11	56.37 ± 13.22	0.849	0.398
Maximal inflation pressure, atm	9.66 ± 2.54	10.23 ± 2.63	−1.280	0.204
LONGTY DCB, n (%)	23 (52.3%)	64 (43.8%)	0.970	0.325

Data are presented as mean ± standard deviation or n (%)

*LAD* Left anterior descending artery; *LCX* Left circumflex artery, *RCA* Right coronary artery; *D* Diagonal branches, *RI* Ramus intermedius artery

### Logistic regression analysis about influencing factors

Logistic regression analysis was conducted on the indicators with statistical significance in the above univariate analysis, as showed by Table 4: Possible influencing factors for target lesions to develop binary restenosis after DCB treatment of ISR are patients with FBG > 6.1 mmol/L (OR: 7185 95% CI: 2.939–17.567 *P* < 0.001) and the length of the lesion (OR:1.046 95% CI: 1.001–1.093 *P* = 0.046). Notably, the longer the lesion length and abnormal levels of FBG, the greater the probability of binary stenosis in the target lesion.

**Table 4:** Logistic regression analysis

	B	SE	Wald	P	OR	95%CI lower	higher
Length of lesion	0.045	0.022	3.973	0.046	1.046	1.001	1.093
Amount of lesion	0.764	0.526	2.112	0.146	2.147	0.766	6.017
Glu > 6.1 mmol/L	1.972	0.456	18.691	< 0.001	7.185	2.939	17.567

*95% CI* 95% confidence interval

## Discussion

CAD is one of the major cardiovascular diseases affecting people worldwide. PCI is an effective treatment for CAD, and with the rapid development of interventional cardiology, many devices like different generation drug-eluting stents and drug-coated balloon are being used in clinical practice [12]. The DCB, as a novel interventional strategy, was first introduced to reduce the restenosis rate of BMS or DES. It can achieve an interventional therapeutic effect without leaving the implant behind, reducing the risk of exotic implantation-associated complications, avoiding multiple stents, and reducing the incidence of thrombosis. Furthermore, the duration of dual antiplatelet therapy is also greatly reduced after DCB treatment [13]. Due to the above advantages, DCB has been increasingly used in coronary intervention, especially in treating ISR. Still the efficacy and safety of DES versus DCB for DES ISR remain to be determined due to the lack of validated randomized controlled trials without intrinsic bias [14]. Recurrent in-stent restenosis refers to the reoccurrence of stenosis after successful treatment of ISR lesions, in which the incidence, influencing factors, and treatment of which are still controversial [15]. A study of predictors associated with recurrent restenosis after sirolimus-eluting stents treatment showed that out of a total of 1965 lesions in 1393 patients, recurrent restenosis occurred in 78 lesions in 66 patients and that smaller minimal lumen diameter at first PCI and acute stent recoil at second PCI are predictors of recurrent restenosis [16]. As a result, we speculated whether recurrent restenosis after DCB for ISR differs from stenting. Therefore, we performed this study to explore possible influencing factors for the recurrent ISR after DCB treatment.

In this study, we have collected data of biological, mechanical and technical factors related to the patient from a multicenter clinical trial and conducted statistical analysis to explore the incidence and possible risk factors of re-restenosis in patients with ISR after DCB treatment. Firstly, we found that the incidence of binary restenosis after 9 months of follow-up was 24.7%, which was relatively high. Secondly, multivessel lesion, the longer length of target lesions, and abnormal fasting blood glucose levels may be risk factors for binary restenosis in ISR patients in this study. It can be seen that in this study, clinical features played a less relevant role in predicting restenosis. In contrast, the lesion characteristics, such as lesion length and the number of target lesions, are associated with the appearance of restenosis.

Some studies have suggested that the length of the stent or lesion is essential for ISR, patients with longer stents or lesions seem to have a higher probability of ISR [17]. Cheng et al. [18] demonstrated that the longer stent is related to severe vascular injury, strong inflammatory reaction, and increased intimal thickness and these findings were consistent with our results. If the lesion length is longer, the lesion area of the intima is larger, the inflammatory response is aggravated, and the blood flow resistance is more significant, all of which are the causes of restenosis. Therefore, these findings remind the surgeon that it is necessary to carefully evaluate the lesion and select the appropriate instrument when treating a patient with long lesions. Meanwhile, some also mention small vessel lesions as a risk factor for ISR. Both BMS and DES performed poorly in treating small vessel disease in the coronary arteries, and the incidence of restenosis when using DES in small vessel lesions is as high as 30%, and the late loss of lumen is significant as well [7]. In this study, when patients were treated with DCB, reference vessel diameter in two groups has no difference. This may be due to no exotic implantation, the small vessels’ original anatomical structure and function of have not been destroyed. However, additional research should be carried out to verify these possibilities. In addition, other studies have revealed that the number of lesions is significantly related to the occurrence of restenosis. After stent implantation, the complications of late stent thrombosis leading to stent restenosis are inevitable. Therefore, the more stents, the greater the risk and damage to blood vessels [19]. Stent implantation can cause damage to endothelial cells’ structure and function, affecting the repair of vascular endothelial and promoting new atherosclerosis. In this study, we also found that patients have a greater chance of developing ISR when having ≥2 target lesions. Other factors related to the lesions, such as the number of calcified lesions and types of target vessels, were not significantly abnormal between the two groups in our study.

Stent under expansion is a common cause of ISR, and it is critical to comprehensively assess the mechanical issues with a stent before the procedure. Intravascular imaging seems to be an effective means of guiding optimal treatment strategies to reduce recurrent restenosis [20]. The delicate procedure is very challenging for surgeons. Many views have indicated that the pre-preprocess with DCB differs from the processing with DES. Sufficient preparation for the lesion is conducive to the full contact and release of the drugs in DCB with the vascular endothelium, which experts recommend in the clinical application of DCB at home and abroad [21, 22]. As such, the following three conditions should be met: no dissection, or A or B type dissection; TIMI blood flow grade III and stenosis should remain ≤30%. Notably, the RIBS IV trial shows that if ISR lesions are pretreated inadequately, the efficacy of DCB treatment is significantly inferior to that of DES [23]. Therefore, pre-dilation is a subjective procedure that depends on the surgeon’s understanding and a wealth of experience [24].

Demographic characteristics and laboratory indicators occupy a large part in many studies on the influencing factors of ISR. However, current studies have not reached a consensus on whether these factors are associated with the occurrence of ISR. The history of smoking, diabetes, and hypertension have been identified as possible risk factors for atherosclerosis and ISR [25, 26]. Some articles have shown that atherosclerosis is significantly increased in patients with hypertension or diabetes, and the possibility of having CAD in these patients is 2 to 4-fold than in normal people [27]..

Notably, hypertension is significantly associated with the risk of coronary artery disease and major cardiovascular events [28]. A retrospective study investigating the role of blood pressure (BP) levels at the time of PCI on the risk of ISR, included 796 patients who had previously received PCI and underwent renewed angiography due to cardiovascular events. It was concluded that normal BP at the time of operation is associated with a nearly 24% risk reduction of ISR, and those with abnormal systolic BP levels tend to have an increase risk of developing ISR. Therefore, excessive blood pressure can cause damage to vascular endothelial cells and promote vascular smooth muscle cell proliferation, leading to ISR [29]. Consequently, actively controlling blood pressure during surgery warrants our attention.

CAD is one of the significant complications of diabetes mellitus (DM). Several studies have pointed out that vessels in patients with diabetes mellitus have an increased risk of restenosis, after controlling for other possible risk factors such as blood pressure, blood lipids, and age, all-cause and cardiovascular mortality in DM was significantly higher than in non-diabetic subjects [30]. In patients with STEMI, pre-procedural hyperglycemia was significantly associated with the incidence of adverse events after PCI, especially in-stent restenosis, regardless of the history of diabetes [31]. Hyperglycemia, insulin resistance, and the increased presence of advanced glycation end products (AGEs) are several key points leading to an increased risk of coronary artery disease in DM patients [32, 33]. Importantly, hyperglycemia can increase the viscosity of red blood cells. In a hyperglycemic environment, a series of reactions such as promoting oxidative stress through mitochondrial superoxide production, synthesis of AGE through the nonenzymatic oxidation of glycoproteins, and reducing NADPH through polyol accumulation can all collaboratively cause damage to endothelial cells [34, 35]. Insulin resistance helps to increase P2Y-receptor-pathway signal, which lead to platelet aggregation and the imbalance of glucose metabolism activates vascular endothelial inflammation, leading to abnormal lipid metabolism, which may be important causes of coronary heart disease and ISR [36, 37]. Compared with non-diabetic patients, neointimal hyperplasia in diabetic patients showed more aggressive phenotypes while the possible cause of ISR is caused by endothelial damage and the proliferation of neointima and vascular smooth muscle cells (VSMC) [38]. In our study, the conclusion that patients with abnormal fasting glucose levels are more likely to develop R-ISR is valid. Therefore, strict preoperative glycemic control is essential for patients who underwent revascularization.

## Conclusion

In this study, we have found that longer lesion lengths, more target lesions, and FBG > 6.1 mmol/L per individual may be characteristics of patients presenting ISR following treatment. Future research should focus on making more innovations in drug-coated balloon for treatment, such as using more auxiliary devices for long and multi-vessel lesions.

### Limitation

The study has some limitations. Firstly, restenosis was evaluated according to quantitative coronary angiography without specifying whether it was combined with intravascular imaging techniques such as IVUS or OCT, making it potentially subject to some error in evaluating the degree of stenosis. Secondly, some patients were unclear about their disease history and were not well documented. Additionally, some patients were likely not examined in the laboratory indicators, resulting in missing data, which may impact our analysis. Thirdly, only about 80% of the patients participated in the 9 months angiography follow-up, and the lack of data for this group of patients who did not participate in the angiographic follow-up may introduce some errors in the data analysis. In the future, more prospective, large-sample, multi-center studies should be conducted to clarify possible factors.

## Data Availability

The datasets used and/or analysed during the current study are available from the corresponding author on reasonable request.

## Abbreviations

DCB: Drug-coated balloon
ISR: In-stent restenosis
FBG: Fasting plasma glucose
PCI: Percutaneous coronary intervention
BMS: Bare-metal stents
DES: Drug-eluting stents
TLR: Target lesion revascularization
MACE: Major adverse cardiovascular event
ECG: Electrocardiogram
CAD: Coronary artery disease
AGEs: Advanced glycation end products
BMI: Body mass index
MI: Myocardial infarction
HF: Heart failure
LVEF: Left ventricular ejection fraction
WBC: White blood cell
N: Neutrophils
RBC: Red blood cell
Hb: Hemoglobin
Plt: Platelet
ALT: Alanine aminotransferase
AST: Aspartate aminotransaminase
ALP: Alkaline phosphatase
TBIL: Total bilirubin
DBIL: Direct bilirubin
TG: Triglycerides
HDL-C: High-density lipoprotein cholesterol
LDL-C: Low-density lipoprotein cholesterol
Cr: Creatinine
Glu: Glucose
SD: Standard deviatio
BP: Blood pressure
